# GMP-Compliant Radiosynthesis of [^18^F]GP1, a Novel PET Tracer for the Detection of Thrombi

**DOI:** 10.3390/ph14080739

**Published:** 2021-07-28

**Authors:** Verena Hugenberg, Marion Zerna, Mathias Berndt, Reinhard Zabel, Rainer Preuss, Dirk Rolfsmeier, Janet Wegener, Henrik Fox, Astrid Kassner, Hendrik Milting, Norman Koglin, Andrew W. Stephens, Jan F. Gummert, Wolfgang Burchert, Marcus-André Deutsch

**Affiliations:** 1 Institute of Radiology, Nuclear Medicine and Molecular Imaging, Heart and Diabetes Center North Rhine-Westphalia, University Hospital Ruhr-University Bochum, Georgstr. 11, 32545 Bad Oeynhausen, Germany; rzabel@hdz-nrw.de (R.Z.); rpreuss@hdz-nrw.de (R.P.); drolfsmeier@hdz-nrw.de (D.R.); jwegener@hdz-nrw.de (J.W.); wburchert@hdz-nrw.de (W.B.); 2 Life Molecular Imaging GmbH, Tegeler Str. 6-7, 13353 Berlin, Germany; m.zerna@life-mi.com (M.Z.); m.berndt@life-mi.com (M.B.); n.koglin@life-mi.com (N.K.); a.stephens@life-mi.com (A.W.S.); 3 Heart Failure Department, Heart and Diabetes Center North Rhine-Westphalia, University Hospital Ruhr-University Bochum, Georgstr. 11, 32545 Bad Oeynhausen, Germany; hfox@hdz-nrw.de; 4 Department of Thoracic and Cardiovascular Surgery, Heart and Diabetes Center North Rhine-Westphalia, University Hospital Ruhr-University Bochum, Georgstr. 11, 32545 Bad Oeynhausen, Germany; jgummert@hdz-nrw.de (J.F.G.); mdeutsch@hdz-nrw.de (M.-A.D.); 5 Department of Thoracic and Cardiovascular Surgery, Erich & Hanna Klessmann Institute, Heart and Diabetes Center North Rhine-Westphalia, University Hospital Ruhr-University Bochum, Georgstr. 11, 32545 Bad Oeynhausen, Germany; akassner@hdz-nrw.de (A.K.); hmilting@hdz-nrw.de (H.M.)

**Keywords:** [^18^F]GP1, GMP compliant radiosynthesis, detection of thrombi, PET/CT imaging, BPVT, LVAD, thrombosis

## Abstract

Thrombus formation and thromboembolic events play important roles in various cardiovascular pathologies. The key receptor involved in platelet aggregation is the fibrinogen receptor glycoprotein IIb/IIIa. [^18^F]GP1, a derivative of the GPIIb/IIIa antagonist elarofiban, is a specific ^18^F-labeled small-molecule radiotracer that binds with high affinity to GPIIb/IIIa receptors of activated platelets. An improved, robust and fully automated radiosynthesis of [^18^F]GP1 has been developed. [^18^F]GP1 has been synthesized with decay corrected radiochemical yields of 38 ± 6%, with a radiochemical concentration up to 1900 MBq/mL, molar activities of 952–9428 GBq/µmol and a radio-chemical purity >98%. After determination of the optimal reaction conditions, in particular for HPLC separation, adaption of the reaction conditions to PET center requirements, validation of the manufacturing process and the quality control methods, the synthesis of [^18^F]GP1 was successfully implemented to GMP standards and was available for clinical application. We describe the GMP-compliant synthesis of the novel radiotracer [^18^F]GP1. Moreover, we provide some proof-of-concept examples for clinical application in the cardiovascular field. PET/CT with the novel small-molecular radiotracer [^18^F]GP1 may serve as a novel highly sensitive tool for visualizing active platelet aggregation at the molecular level.

## 1. Introduction

Thrombus formation and thromboembolic events play important roles in various vascular pathologies such as deep vein thrombosis, stroke or pulmonary embolism [1]. The deposition of circulating platelets is a hallmark component of thrombus formation. The key receptor involved in platelet aggregation is the fibrinogen receptor glycoprotein IIb/IIIa, also known as αIIbβ3, which is a member of the integrin family of cell surface proteins and is expressed specifically and in high density on activated platelets. Stimulation and activation of the platelet by a variety of agents (e.g., thrombin, adenosine diphosphate, thromboxane) causes conformational changes and allosteric activation of GPIIb/IIIa receptor resulting in the exposure of its extracellular containing ligand-binding domain. During the early phase of activation additional GPIIb/IIIa, receptors externalize to the platelet surface. The KQAGDV amino acid motif in the C-terminus of fibrinogen is required for binding to GPIIb/IIIa and selectively binds the active conformation [2]. Platelet aggregation is mediated by the GPIIb/IIIa receptor (integrin αIIbβ3), one of the most abundant cell surface receptors (≈80,000 per platelet), which represents ≈ 15% of total surface protein. [^18^F]GP1, a derivative of the GPIIb/IIIa antagonist elarofiban, is a specific ^18^F-labeled small-molecule radiotracer that binds with high affinity to GPIIb/IIIa receptors of activated platelets (IC_50_ = 20 nM) and was designed for positron emission tomography (PET) imaging of thrombotic material. Elarofiban was selected as a scaffold for the development of [^18^F]GP1 based on its high affinity to GPIIb/IIIa receptors of activated platelets, a very high specificity for binding GPIIb/IIIa over other closely related integrins (300,000-fold specificity for GPIIb/IIIa over αvβ3), its suitable pharmacokinetic properties, and the feasibility for incorporating the ^18^F-radiolabel without losing biological activity [3]. A favorable safety, pharmacokinetic and biodistribution profile as well as excellent diagnostic performance of [^18^F]GP1 PET/CT for the detection of acute venous and arterial thromboembolism has recently been demonstrated [4,5]. In an open-label, non-randomized, first-in-human phase 1 study [^18^F]GP1 PET/CT identified thromboembolic foci in all patients with either acute deep vein thrombosis (DVT) or pulmonary embolism (PE). The tracer was well tolerated without any drug-related adverse events and showed high initial uptake in spleen, kidney and blood pool, followed by rapid clearance [6]. Within this report, we describe the optimization of the synthesis of the small-molecule radiotracer [^18^F]GP1 and the implementation to GMP standard for routinely clinical production at the Heart and Diabetes Center North Rhine-Westphalia (HDZ NRW). Furthermore, we show that—beyond its application in DVT and PE—[^18^F]GP1 may also be clinically useful in detecting thrombotic material in left ventricular assist devices (LVAD) and bioprosthetic valve thrombosis.

## 2. Results and Discussion

### 2.1. Optimization of the [^18^F]GP1 Synthesis

The radiosynthesis of [^18^F]GP1 was first reported 2017 by Lohrke et al., giving only few details about its practical realization. [^18^F]GP1 was synthesized via two-step procedure starting with the direct nucleophilic substitution of the Boc-protected tosylate precursor ([^18^F]fluoride, K_2_CO_3_, K_222_, 120 °C, 8 min), followed by acidic deprotection (2 N HCl, 110 °C, 4 min) and HPLC purification [7].

Adaption and optimization of reaction conditions and supplementation of the parameters that were not described in detail in the publication by Lohrke et al. finally led to the successful implementation of a fully automated GMP compliant synthesis for [^18^F]GP1. Reaction times, temperatures and amounts of reagents were adjusted. The evaporation of HCl was avoided due to incompatibility of hot cell, compression plant and heat recovery system with corrosive gases. The deprotection reaction with aqueous HCl was accomplished in the closed reactor, and the reaction mixture was neutralized with aqueous NaOH solution before transfer to the HPLC system. HPLC purification proved to be difficult due to the zwitterionic character of the GP1 molecule. Here, ensuring a consistent pH during the HPLC purification is essential in order to enable sufficient separation of the product from the different GP1 related side products. Solvent mixtures of water/acetonitrile containing 1% trifluoroacetic acid (e.g., 10/90/0.1) did not lead to a sufficient separation. Therefore, a glycine buffer (10 mM), which was set to pH 10, was used to stabilize the pH during the HPLC purification. A mixture of 14% ethanol in glycine buffer (10 mM, pH 10) turned out to be the best solvent for a sufficient HPLC separation. The final product is highly prone to undergo defluorination due to radiolysis. To avoid radiolysis, [^18^F]GP1 was formulated in ascorbic acid solution (27.3 mg/mL).

### 2.2. Clinical Production of [^18^F]GP1

**Scheme 1:**
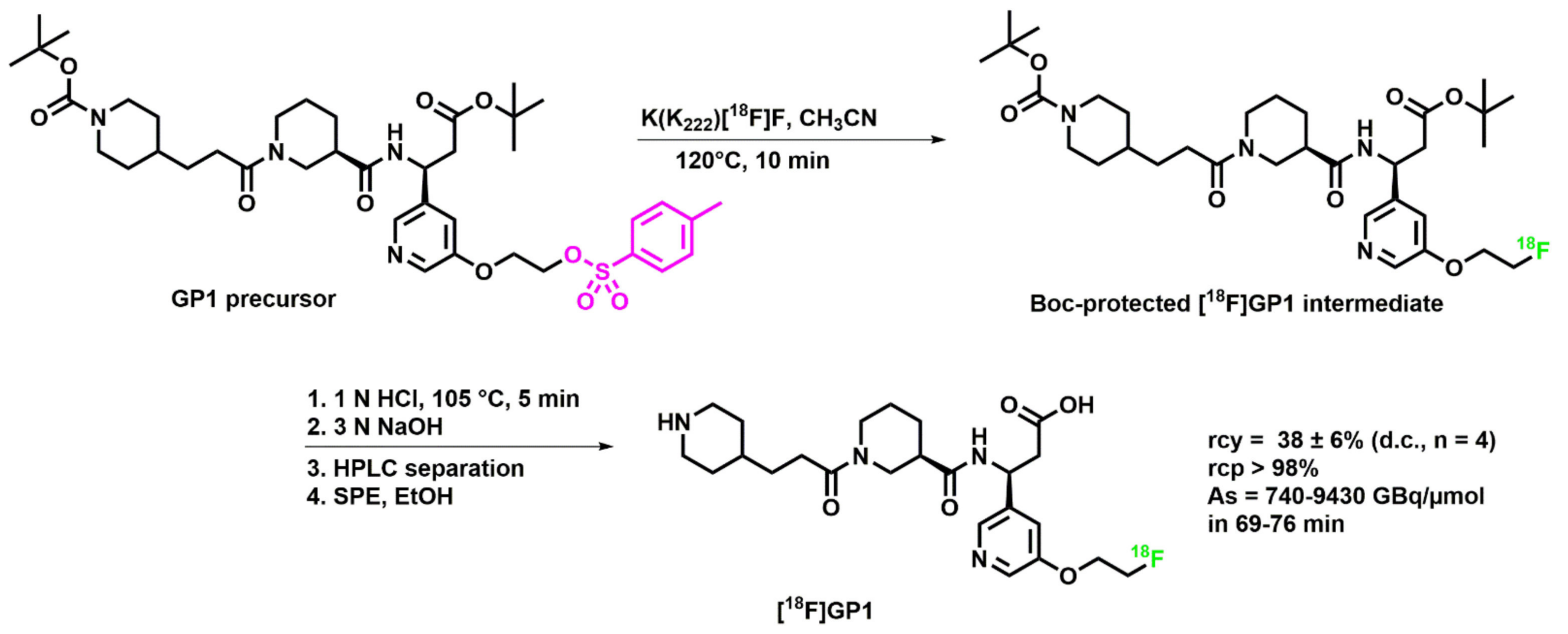
Synthesis of [^18^F]GP1.

The GMP compliant automated synthesis of [^18^F]GP1 for clinical production was accomplished as described below (Scheme 1). In a computer controlled EZAG Modular-Lab EC-187 synthesizer aqueous [^18^F]fluoride ions (60–100 GBq) from the cyclotron target were passed through an anion exchange resin (Sep-Pak^®^ Light Waters Accell™ Plus QMA cartridge, preconditioned with 10 mL of 0.5 M K_2_CO_3_ and 10 mL of water for injection (WFI)). [^18^F]Fluoride ions were eluted from the resin with a Kryptofix^®^2.2.2 (K_222_)/K_2_CO_3_ solution (1.0 mg K_2_CO_3_ and 5 mg K_222_ dissolved in 250 μL of WFI and 1250 μL of DNA-grade acetonitrile) in the reactor. Subsequently, the aqueous K(K_222_)[^18^F]F solution was carefully evaporated to dryness in vacuo. An amount of ~7.0 mg (~8.9 µmol) of Boc-protected precursor (GP1 precursor) dissolved in 0.5 mL anhydrous acetonitrile was added to the dried K(K_222_)[^18^F]F residue and the reaction mixture was heated at 120 °C for 10 min. After cooling to 80 °C, 1 mL of 1 N aqueous HCl was added, and the reaction mixture was heated at 105 °C for 5 min. Cooling to 80 °C was followed by neutralization with 3 N aqueous NaOH (250 µL) and 3 mL of WFI, transfer to the HPLC loop via vented sterile filter and injection on the HPLC column. The product peak was collected at retention times of ~11–14 min. The collected solvent was diluted with 70 mL of WFI and transferred to a Waters Sep-Pak Plus Short tC18 cartridge (preconditioned with 10 mL of ethanol and 10 mL of WFI). The cartridge was washed with additional 6 mL of WFI and eluted with 1.5 mL of ethanol into the product vial with 16.5 mL of sodium ascorbic acid solution (27.3 mg/mL). Using nitrogen pressure, the product solution was passed from the product vial through a 0.22 µm sterile filter into a sterile, filter-vented final product vial. The radiosynthesis provided [^18^F]GP1 with an overall radiochemical yield of 32–44% (based on cyclotron-derived [^18^F]fluoride, d. c., n = 5) in 68–76 min from end of radionuclide production. [^18^F]GP1 was isolated in radiochemical purities of > 98 % and a molar activity in the range of 950–9430 GBq/µmol at the end of radiosynthesis. Radiochemical purities and molar activities were determined using analytical radio-HPLC system (see Section 3.2, retention time of R_t_ [^18^F]GP1 = 6.5 ± 0.7 min).

Determination of the shelf-life of [^18^F]GP1 injection solution with a high radioactivity concentration of ~1900 MBq/mL revealed high stability of [^18^F]GP1 over a period of 8 h from end of synthesis (EOS). No defluorination occurred, and the specifications for radiochemical purity (>90% [^18^F]GP1) and pH were still met. 

The synthesis process, as well as the analytical HPLC and GC methods, were validated. Results of the process validation batches are presented in Table 1. The process was considered to be appropriate for [^18^F]GP1 routine production.

The successful implementation of the fully automated GMP compliant synthesis enabled the delivery of [^18^F]GP1 for clinical use. At HDZ NRW, [^18^F]GP1 was investigated for the detection of thrombi in patients when conventional imaging methods such as CT, echocardiography or ultrasound were not able to provide reliable information on thrombus formation. Applications include the detection of thrombi in LVADs and the diagnosis of bioprosthetic valve thrombosis.

### 2.3. Proof-of-Concept Examples for Clinical Applications

Bioprosthetic valve thrombosis (BPVT) is an important clinical entity eventually following both bioprosthetic surgical and transcatheter aortic valve replacement. Although the thrombogenicity of bioprosthetic heart valves appears to be much lower than in mechanical heart valves, recent studies show that valve or leaflet thrombosis following bioprosthetic surgical (SAVR) or transcatheter aortic valve replacement (TAVR) is not an infrequent observation. The continuing debate regarding optimal post-implant antithrombotic regimens and ongoing evaluations of the performance and durability after both bioprosthetic SAVR and TAVR has increased the awareness for this phenomenon. BPVT can be symptomatic or subclinical. Depending on the imaging modality utilized and diagnostic criteria employed, the incidence of BPVT ranged from as low as 0.6% and up to 40% [8,9,10,11,12,13]. Of note, several recent reports suggest that the incidence of subclinical BPVT is considerably higher [14,15]. Transthoracic echocardiography (TTE) is the first-line screening tool for patients with suspected thrombosis and or prosthetic valve hemodynamic deterioration. If TTE is inconclusive, additional transesophageal echocardiography (TEE) imaging is recommended. Echocardiography can visualize thrombotic structures on valve leaflets and is used to determine the degree of valve obstruction by measuring transprosthetic gradients. Because acoustic shadowing caused by the stent frame of the prosthesis may limit visualization of thrombotic material, the diagnostic accuracy of echocardiography may be suboptimal.

**Figure 1:**
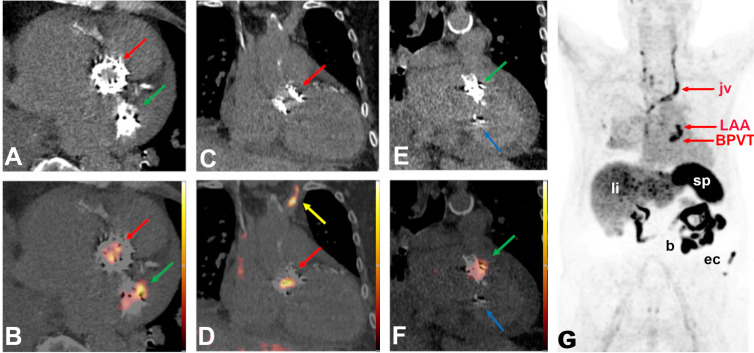
[^18^F]GP1 PET/CT imaging findings of patient #1 with BPVT and thrombus in left atrial appendage (LAA) adherent to LAA occluder. (**A**) Axial view of unenhanced low-dose CT used for attenuation correction and anatomical landmarking with BPVT (red arrow) and thrombus in LAA (green arrow). (**B**) Axial view of corresponding fusion PET/CT images with [^18^F]GP1, BPVT (red arrow), thrombus in LAA (green arrow). (**C**) Coronal view of unenhanced low-dose CT with BPVT (red arrow). (**D**) Coronal view of the corresponding fusion PET/CT images with [^18^F]GP1, BPVT (SUVmax 7.1, red arrow) and thrombi in jugular vein (SUVmax 9.7, yellow arrow). (**E**) Parasagittal view of unenhanced low-dose CT with thrombus in LAA (green arrow). (**F**) Parasagittal view of corresponding fusion PET/CT image with [^18^F]GP1 with LAA thrombus (SUVmax 6.8, green arrow). No tracer accumulation was seen at the MitraClip device (blue arrow). (**G**) Maximum intensity projection (MIP) of [^18^F]GP1 PET showing physiologic tracer distribution within visceral organs and clearly visible tracer accumulation at the site of aortic valve replacement (BPVT). Additional tracer accumulation can be observed within the LAA as well as in the course of the left jugular vein (jv). Abbreviations: b—bowel, BPVT—bioprosthetic valve thrombosis, ec—external contamination, jv—jugular vein, LAA—left atrial appendage, li—liver, sp—spleen. Color bars representing SUVs from 0–5.12 bw.

Patient #1 is a 76-year-old male patient who had undergone transfemoral TAVR with a 26 mm bioprosthesis (Edwards Sapien, Edwards Lifesciences Corp. Irvine, CA, USA) for decompensated severe aortic valve stenosis and severely reduced left ventricular ejection fraction in March 2012. In 2019, the patient underwent mitral valve clipping for severe functional mitral regurgitation. In the same year, percutaneous left atrial appendage occlusion with an Amplatzer Amulet device was performed after the patient developed gastrointestinal bleeding episodes under oral anticoagulation, which he had taken for atrial fibrillation and the prevention of thromboembolism. In August 2020, the patient presented with severe prosthetic valve hemodynamic deterioration (mean pressure gradient 55 mmHg, effective orifice area 0.6 cm² and moderate aortic regurgitation). Computed tomography angiography revealed severely calcified prosthetic valve leaflets typical for bioprosthetic valve degeneration. Eventually, the patient was scheduled for valve-in-valve TAVR with a balloon-expandable bioprosthesis (Edwards Sapien 3, size 26 mm). Four days after a primarily successful and uncomplicated procedure, routine transthoracic echocardiography revealed an acceleration of peak aortic velocity and an increased mean transprosthetic gradient of 4.1 m/s and 35 mmHg, respectively, with a corresponding aortic valve area of 1.2 cm² as calculated by the continuity equation. Because of thickened and restricted bioprosthetic valve leaflets with visible apposition of echogenic material, bioprosthetic valve thrombosis (BPVT) was suspected. [^18^F]GP1 PET/CT was performed to confirm the diagnosis of BPVT. [^18^F]GP1 PET/CT clearly distinguished between blood pool activity and thrombotic foci (Figure 1). The SUVmax in thrombotic foci at the bioprosthesis and the left atrial appendage were 7.1 and 6.8 at 90 min post-injection.

After heart team discussions and in accordance with the current ESC/EACTS guideline recommendations (Class I, level of evidence C), oral anticoagulation with the Vitamin K antagonist phenprocoumon was initiated.

Although dynamic 4-dimensional multidetector computed tomography (4D-MDCT) has greatly improved the diagnosis of bioprosthetic valve thrombosis (BPVT), more sensitive and pathology-specific non-invasive imaging tools are lacking. Recently, the glycoprotein IIb/IIIa receptor targeted, elarofiban-derived PET/CT imaging radiotracer [^18^F]GP1 has been successfully used for visualization of acute venous and arterial thrombi. [^18^F]GP1 PET/CT may overcome some limitations of current diagnostic imaging modalities for detecting BPVT and may prove useful for monitoring of therapeutic approaches. Recent observational data have suggested that bioprosthetic durability may be enhanced by concomitant anticoagulation therapy [16]. Therefore, BPVT may hypothetically act as a potential trigger to valve degeneration that might be prevented through early detection and anticoagulation. Further, this method may be a suitable alternative to 4D-MDCT in patients with impaired renal function or contrast allergy in whom contrast agent administration is contraindicated and does not depend on a low heart rate as 4D-MDCT, respectively. [^18^F]GP1 PET/CT may allow for highly sensitive detection of subclinical BPVT and provide important insights into the pathogenesis of early stages of bioprosthetic valve degeneration [17].

Mechanical circulatory support (MCS) with left ventricular assist devices (LVAD) is a safe and efficacious treatment strategy for patients with end-stage heart failure (HF) that is refractory to medical therapy [18,19]. Unimproved, hemostatic complications are frequently observed and dreaded complications in patients with LVADs [20,21]. Despite ongoing efforts to engineer the LVAD surface less thrombogenic as well as the use of aggressive anticoagulation regimens, thrombotic complications continue to affect long-term outcomes post-implant with rates of 7% to 16% per year. Strokes, another debilitating complication post LVAD implantation, are a consequence of thrombus formation and embolic events, occur with an incidence ranging from 7% to 10% per year according to meta-analyses [21]. Studies consistently show that about 50% of strokes in this setting are embolic, and 50% are hemorrhagic. Its occurrence is associated with significant morbidity and mortality [22]. Pump thrombosis denotes the development of clot formation within the flow inside all components that constitute the pump, including the titanium inflow cannula, the outflow graft and the pump housing that contains the rotor itself. The pathophysiologic mechanisms involve complex interactions between device surface, sheer stress, decreased or turbulent blood flow and suboptimal anticoagulation [18]. Timely recognition and urgent treatment are crucial to prevent the reoccurrence of thromboembolic complications [20]. Although complementary use of CT and TTE for evaluation of suspected device malfunction has improved, accurate and reliable identification of LVAD-related complications compared to either modality alone and more sensitive diagnostic modalities are lacking. Metal-related artifacts and acoustic shadowing of the titanium-housing of LVADs significantly impair image quality and decrease the diagnostic value of current imaging techniques.

Patient #2 is a 63-year-old male patient who had undergone LVAD implantation (HeartWare^®^-HVAD, Medtronic) for ischemic end-stage heart failure with severely reduced left ventricular ejection fraction (LVEF 15%) in 2020 due to large anterior acute ST-elevation myocardial infarction. A half year later, the patient was readmitted emergently with recurrent low LVAD flow alarms. LVAD thrombosis was suspected through HVAD logfile readouts (low flow, power spikes) and when “third-harmony” (rotor vibrates with a frequency three times the rotational speed of the pump) occurred in the logfile, which is an established bioacoustics parameter suspecting impeller imbalance. [^18^F]GP1 PET/CT was performed to rule out thrombosis of the LVAD system components.

**Figure 2:**
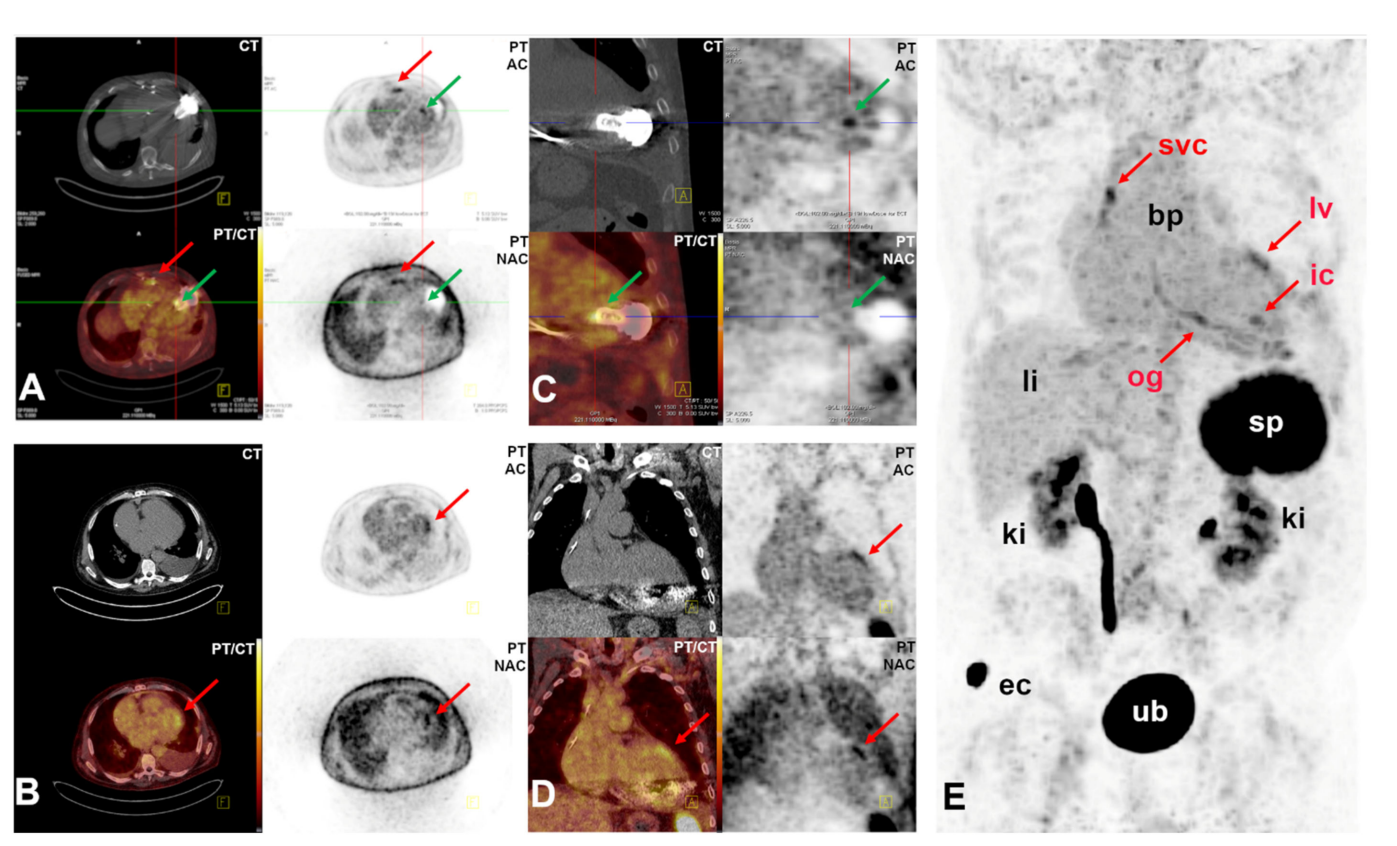
[^18^F]GP1 PET/CT imaging findings of patient #2 with small thrombi in inflow cannula and outflow graft of the LVAD and residuum of thrombus adherent to the anterior wall. (**A**) Axial views of CT, PET attenuation corrected (PT AC), PET non-attenuation corrected (PT NAC) and corresponding fusion PET/CT images showing the small thrombi in inflow cannula (SUVmax 4.9, green arrows) and outflow graft (SUVmax 4.3, red arrows). (**B**) Axial views CT, PT AC, PT NAC and PT/CT images showing the small thrombus adherent to the anterior wall (SUVmax 5.5, red arrows). (**C**) Coronal views of CT, PT AC, PT NAC and PT/CT images showing the small thrombus in the inflow cannula (red arrows). (**D**) Coronal views of CT, PT AC, PT NAC and PT/CT images showing the small thrombus adherent to the anterior wall. (**E**) Maximum intensity projection (MIP) of [^18^F]GP1 PET showing physiologic tracer distribution within visceral organs and visible tracer accumulation in inflow cannula (ic) and outflow graft (og) of the LVAD, adherent to the anterior wall (lv) and in the superior vena cava, located at the tip of central venous catheter (SUV max 6.0, svc). Abbreviations: bp—blood pool, ec—external contamination, ic—inflow cannula, ki—kidney, li—liver, lv—left ventricle, og—outflow graft, svc—superior vena cava, ub—urinary bladder. Color bars representing SUVs from 0–5.12 bw.

[^18^F]GP1 PET/CT visualized activity accumulation near and around the inflow cannula of the LVAD as well as in the area of the outflow graft (Figure 2). With the diagnosis of HVAD thrombosis confirmed, systemic lysis therapy with t-PA was successfully applied under close vital sign monitoring on the intensive-care unit. Eventually, the patient could be discharged with normalized LVAD function parameters (flow, power and pulsatility indices). With the clinical example in this report, we show that [^18^F]GP1 PET/CT can visualize even smaller regions of active platelet aggregation.

### 2.4. In Vitro Experiments

Prosthetic valve dysfunction may be provoked by reduced leaflet motion or impaired leaflet coaptation. Four main etiologies with different clinical implications may account for prosthetic valve hemodynamic deterioration: (1) thrombosis, (2) fibrotic pannus ingrowth, (3) structural degeneration and (4) prosthetic valve endocarditis with vegetation formation [8,23]. For further therapeutic decision-making it is important to differentiate clearly between these clinical entities which can be challenging in some instances. The association of coagulation with infective endocarditis has been well established. Increasing evidence indicates that many bacterial pathogens use similar mechanisms to mediate platelet activation. Initial adhesion of bacteria to platelets occurs either directly or, more commonly, through a plasma protein bridge with a platelet receptor, usually GPIIb/IIIa or GPIb [24].

**Figure 3:**
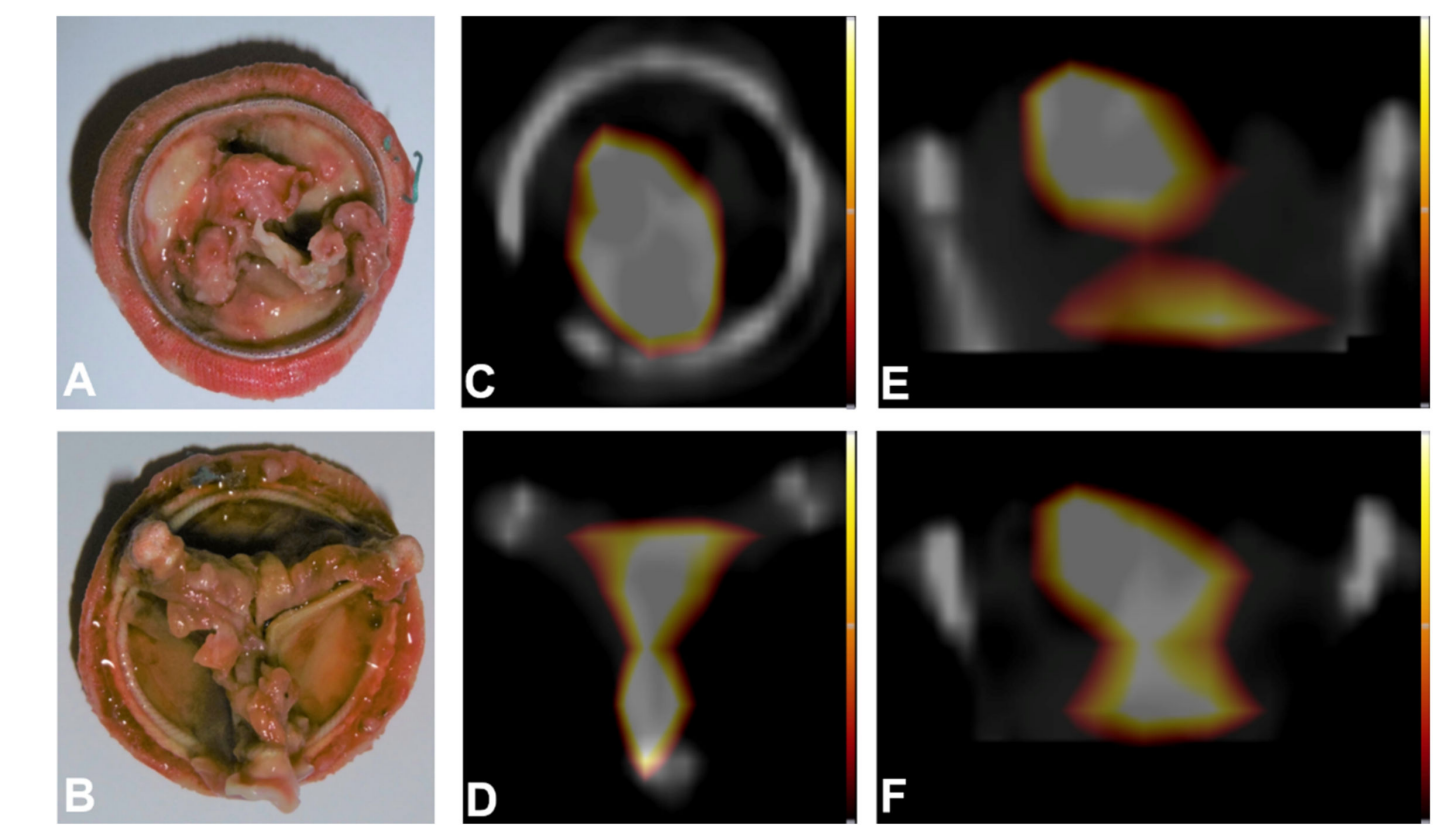
In vitro experiment with explanted bovine pericardial aortic bioprosthesis. (**A**) Bottom view of explanted bioprothesis showing extensive endocarditic vegetation, (**B**) Top view of explanted bioprosthesis, (**C**) Fused PET/CT bottom view, (**D**) Fused PET/CT top view, (**E**) Fused PET/CT, side view, (**F**) Fused PET/CT lateral view. Color bars representing activity concentrations from 1–31,336 Bq/mL.

**Figure 4:**
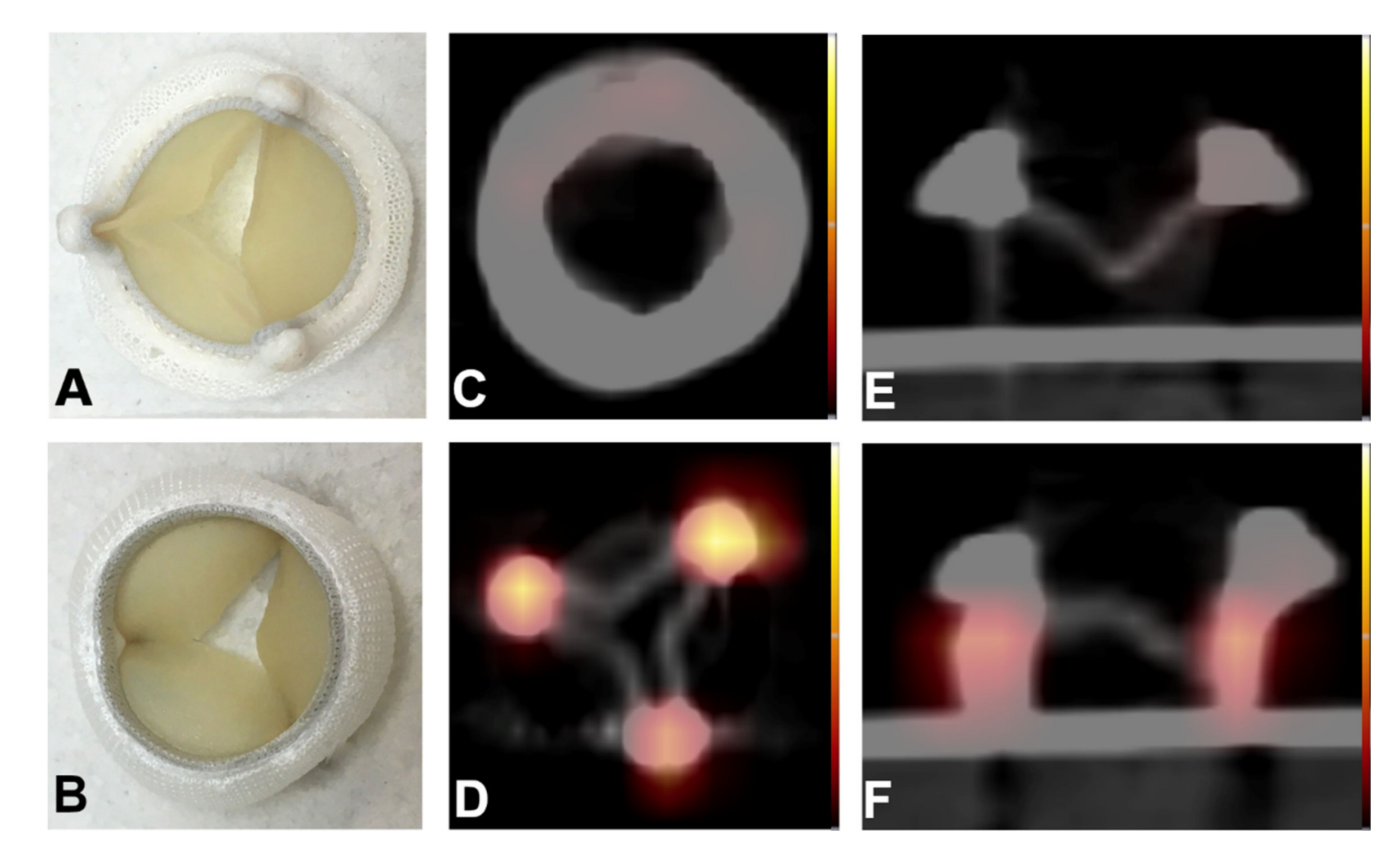
Unused bioprosthesis. (**A**) Bottom view of bioprosthesis, (**B**) Top view of bioprosthesis, (**C**) Bottom aspect of fused PET/CT—no accumulation, (**D**) Top view of fused PET/CT showing tracer accumulation on stent posts, (**E**) Lateral view of bioprosthesis (center) showing no tracer accumulation on leaflets (fused PET/CT), (**F**) Lateral view of bioprosthesis showing tracer accumulation on stent posts (fused PET/CT). Color bars representing activity concentrations from 4–511 Bq/mL.

For in vitro assays, a freshly explanted bovine pericardial aortic bioprosthesis extensively covered with endocarditic vegetations was used. The 46-old male patient from which the valve prosthesis had been taken had previously undergone surgical aortic valve replacement with a bioprosthesis (Edwards Perimount Aortic, model 2900, size 27mm) for severe aortic valve stenosis in 2019. He now suffered from infective prosthetic valve endocarditis which was accompanied by an aortic root abscess. One day before emergent admission, the patient presented with right middle cerebral artery stroke and left common femoral artery occlusion at the referring hospital. The patient underwent emergent redo aortic valve replacement with reconstruction of the aortic root. Within blood cultures as well as in specimen taken from the valve during surgery, Enterococcus faecalis was identified as the responsible microorganism. Figure 3 shows the aortic (panel A) as well as the ventricular side (B) of the explanted bioprosthesis. Extensive endocarditic vegetations covering the central portion of the prosthetic valve leaflets can be seen. The explanted bioprosthesis was incubated in [^18^F]GP1/0.9% NaCl solution (20 kBq/mL) for 5 min at room temperature, washed sufficiently with isotonic NaCl solution and investigated via PET/CT. Extensive activity accumulation was observed on the valve leaflets, reflecting the extensive endocarditic vegetation (Figure 3). The endocarditic vegetation on the bioprosthesis was analyzed by western blot using an HRP-labeled antibody against CD41, confirming the presence of the GPIIB/IIIa receptor (Figure S1). An unused Edwards Perimount, size 23 mm, was used as control (Figure 4). Here, no [^18^F]GP1 activity accumulation on the valve leaflets has been observed in PET/CT measurement after incubation with [^18^F]GP1/0.9% NaCl solution (20 kBq/mL) and sufficient washing. Some minor activity accumulation was found in the tips of the three stent posts of the bioprosthesis, due to remaining [^18^F]GP1 solution in suture cuff material. This accumulation was not specific and could probably have been removed by further washing of the prosthesis. The remaining suture cuff and, more importantly, valve leaflets made from bovine pericardium did not show tracer uptake.

While 4D-MDCT scanning can differentiate between BPVT and fibrotic pannus ingrowth only based on Hounsfield units, with BPVT having lower attenuation than fibrotic pannus ingrowth, [^18^F]GP1 PET/CT may reliably discriminate between BPVT and fibrotic pannus ingrowth. However, as demonstrated in Figure 3, [^18^F]GP1 PET/CT may not adequately discriminate between BPVT and the formation of thrombotic vegetations in the context of prosthetic valve endocarditis. Thus, if there is any clinical suspicion for prosthetic valve endocarditis additional FDG-PET may help to further differentiate between infective endocarditis-related thrombus formation and BPVT.

## 3. Materials and Methods

### 3.1. Chemicals and Materials

The synthesis of [^18^F]GP1 was implemented in an automated Modular-Lab EC-0187 synthesis module (Eckert & Ziegler—Berlin, Germany) for routine production. The precursor compound *tert*-butyl 4-{3-[(3*R*)-3-({(1*S*)-3-tert-butoxy-1-[5-(2-{[(4-methylphenyl)sulfonyl]oxy}ethoxy)pyridin-3-yl]-3-oxopropyl}carbamoyl)piperidin-1-yl]-3-oxopropyl}piperidine-1-carboxylat (GP1 precursor) and the GP1 reference compound (*S*)-3-[5-(2-(fluoroethoxy)pyridin-3-yl]-3-[({(3*R*)-1-[3-(piperidin-4-yl)propanoyl]piperidine-3-yl}carbonyl)amino]propanoic acid were acquired from ABX Advanced Biochemical Compounds (Radeberg, Germany). 1,10-Diaza-4,7,13,16,21,24-hexaoxabicyclo[8,8,8]-hexacosane (Kryptofix^®^2.2.2, K_222_), potassium carbonate (K_2_CO_3_, Ph. Eur.), hydrochloric acid ultrapure 30%, acetonitrile (anhydrous, 99.8%), sodium hydroxide (Ph. Eur.), (+)-sodium L-ascorbate (BioXtra, 99.0 %) ethanol (Ph. Eur.) were obtained from Merck (Darmstadt, Germany) or Sigma-Aldrich Biochemie GmbH—A division of Merck KGaA (Hamburg, Germany). Sterile water (WFI, aqua ad iniectabilia, i.v.), syringes and cannula were obtained from B. Braun (Melsungen, Germany), nitrogen from Linde (Germany), sterile filters Cathivex-GV, 0.22 µm and Millex GV, 0.22 µm from Merck Millipore (Burlington, MA, USA). Solid-phase extraction (SPE) Sep-Pak Plus Short tC18 cartridges and Sep-Pak^®^ Light Waters Accell™ Plus QMA cartridges were obtained from Waters Corp. (Milford, MA, USA). All reagents and materials were used as received from the commercial suppliers.

### 3.2. Chromatographic Methods

**Figure 5:**
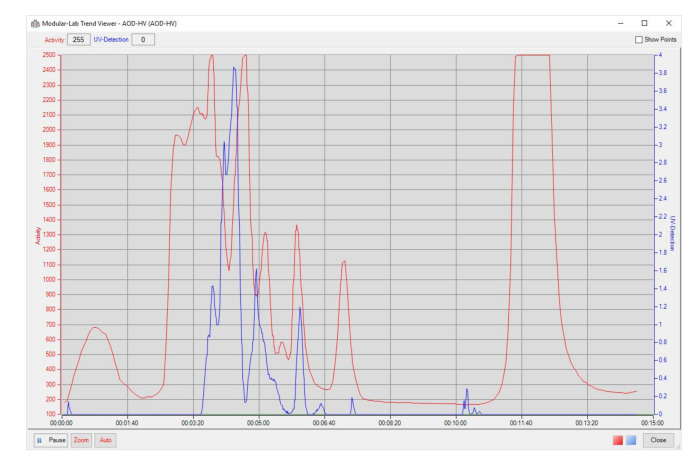
Example diagram for the HPLC separation in the preparation of [^18^F]GP1with a starting activity of ~100 GBq. The UV channel is shown in blue, the gamma channel in red. The product peak is between 11–14 min.

Separation of the radiosynthesized compound was performed on the following semi-preparative radio-HPLC system (λ = 287 nm): S 1122 HPLC pump, smartline UV detector 200 (Knauer GmbH), γ-detector EZAG Modular-Lab EC-187 SD5-109 (Eckert & Ziegler Eurotope GmbH, Berlin, Germany) and an ACE C18 HL HPLC column (5 µm, 250 × 10 mm). The recorded data were processed by the Software Modular-Lab (Eckert & Ziegler Eurotope GmbH, Berlin). Isocratic elution with 10 mM glycine buffer (14% ethanol, pH 10) over 20 min and a flow rate of 5 mL/min was used. Retention time of [^18^F]GP1 was 11 to 14 min (see Figure 5).

Analytical radio-HPLC (λ = 287 nm) was performed using a SL 25 quaternary gradient pump, S 3245 UV/Vis detector, S 3700 γ-detector (Sykam Chromatographie Vertriebs GmbH, Fürstenfeldbruck) and a Phenomenex Gemini column (5 µm, C18, 110 Å, 250 mm × 4.6 mm). The recorded data were processed by the Chromstar software (Sykam Chromatographie Vertriebs GmbH, Fürstenfeldbruck). The HPLC method started isocratic with 10% acetonitrile in water (0.1% TFA) for 15 min, followed by a linear gradient from 10% to 100% acetonitrile in water (0.1% TFA) over 0.5 min, holding for 2.5 min, followed by a linear gradient from 100% to 10% acetonitrile (0.1% TFA) over 0.5 min and holding for 2.5 min, with a flow rate of 1.5 mL·min^−1^. Retention time of [^18^F]GP1 was 6.5 ± 0.7 min.

Different methods and solvent compositions for the preparative and analytical HPLC were used. The pH plays an important role during the purification of the crude product solution. Therefore, for semi-preparative HPLC purification the glycine buffer with a stable pH was used. After formulation the pH of the final product solution does not differ that much and an analytical HPLC method is applicable using eluents like water/acetonitrile/TFA (90/10/0.1).

### 3.3. Radiochemistry

Absolute radioactivity measurements were performed using a calibrated ionization chamber (Isomed 2010, MED Nuklear-Medizintechnik GmbH, Dresden, Germany).

#### 3.3.1. Synthesis module

The synthesis of [^18^F]GP1 is carried out in a shielded hot cell on the automated synthesis module EZAG Modular-Lab EC-187 (Eckert & Ziegler Eurotope GmbH, Berlin). The synthesis unit represents a closed system. The vents of the apparatus are sealed with sterile filters with a pore size of 0.22 µm.

The computer-controlled synthesis process takes place fully automatically according to the specified time lists of the program and is thus completely reproducible. The temperature as a reaction parameter is actively controlled by a feedback. This automatism is used as in-process control (IPC) at the key points of synthesis. Temperature variations of ±1 °C are tolerated as an allowable deviation. During the synthesis, the temperature and radioactivity profiles in the reactor are continuously recorded and documented online. During the HPLC purification, the pressure, radioactivity and UV channels are recorded. In the batch report, the audit trail of the process with all in-process controls and switching of all valves, as well as the synthesis progress curves for temperature and radioactivity in the reactor, and pressure, gamma and UV channel of the HPLC run are documented.

#### 3.3.2. Production Procedures

**Figure 6:**
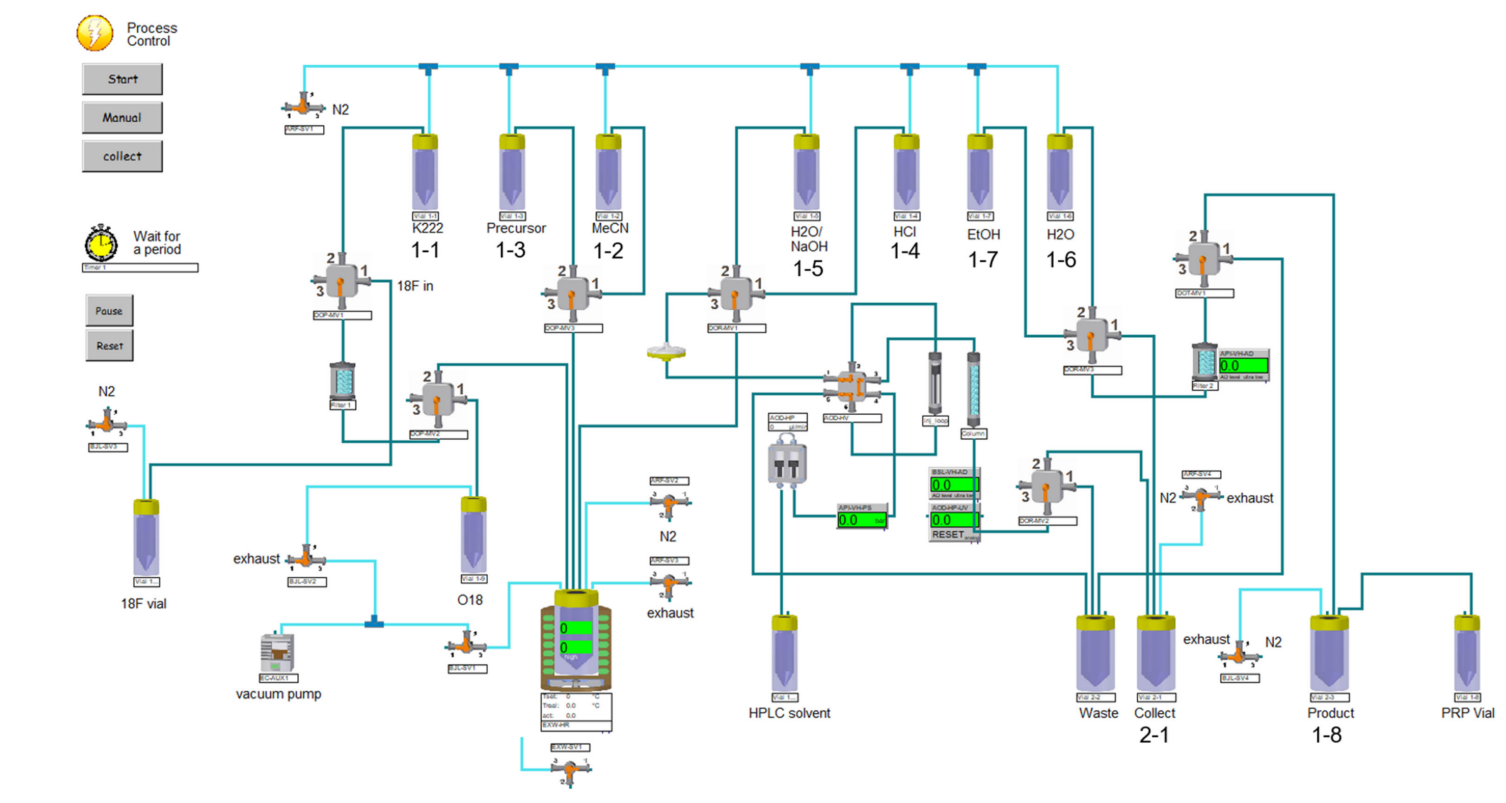
Schematic overview of the EZAG module surface for the synthesis of [^18^F]GP1.

Before every synthesis, the synthesis device has to be cleaned and dried following the device-specific instructions. If the last use was more than one day ago, the module has to be dried again before the next use. All substances are provided in appropriately labeled vessels and used directly for module filling. The cartridges are conditioned (Sep-Pak^®^ Light Waters Accell™ Plus QMA: 10 mL of 0.5 M potassium carbonate solution, 10 mL of water for injection; Sep-Pak Plus Short tC18: 10 mL of EtOH, 10 mL of water for injection) and placed on the corresponding cartridge holders. The storage vials are filled under inert gas pressure with the prescribed quantities of the sterile solutions (see Table 2 and Figure 6). End product filtration into a sterile pyrogen free glass vial is performed via Cathivex^®^ GV sterile filter (0.22 µm) and a Millex GV, 0.22 µm for ventilation.

##### 3.3.3. [^18^F]Fluoride Production

No-carrier-added aqueous [^18^F]fluoride is produced on a GE PETtraceTM 800 (GE Healthcare) cyclotron and an IBA Cyclone 18/9 cyclotron (IBA RadioPharma Solutions) by irradiation of a 2.4 mL water target using 16.5 MeV or 18 MeV proton beams on >97.0% enriched [^18^O]water by the ^18^O(p,n)^18^F nuclear reaction. The beam current is maximum 64 µA. The aqueous [^18^F]fluoride solution (50–100 GBq) is transferred through a PP line by helium overpressure of the target from the cyclotron directly into the collection vial “18F vial” of the synthesis module in the hot cell.

##### 3.3.4. [^18^F]GP1 Production

The automated synthesis was controlled and monitored from the computer screen and comprised the following automated steps:

1. Transfer of the aqueous [^18^F]fluoride to “18F vial”.
2. Trapping of [^18^F]fluoride on the QMA ion exchange cartridge.
3. Elution from the QMA cartridge with K_222_/K_2_CO_3_ solution into the reactor.
4. Evaporation of the solvent in the reactor at 125 °C under nitrogen flow and vacuum.
5. Addition of acetonitrile from vial 1-3. Evaporation at 125 °C under nitrogen flow and vacuum.
6. Cooling of the reactor to 80 °C. Addition of the precursor in anhydrous acetonitrile from vial 1-2.
7. Heating the reaction mixture in the reactor at 120 °C with closed exhaust for 10 min.
8. Cooling the reactor to 80 °C, addition of HCl from vial 1-5.
9. Heating at 105 °C for 5 min with closed exhaust.
10. Cooling the reactor to 80 °C. Addition of WFI/NaOH from vial 1-4.
11. Transfer of the reaction mixture through the vented Cathivex^®^-filter to be loaded onto the HPLC loop.
12. Injection of the loop content onto the HPLC column and elution with 86% 10 mM glycine buffer pH 10.0/14% ethanol at 5 mL/min.
13. Manual collection of the product fraction in vial 2-1 pre-loaded with 70 mL WFI.
14. Transfer of the resulting mixture from vial 2-1 through the solid phase extraction cartridge (tC18 Plus short, Sep-Pak).
15. Rinsing of the SPE with 5 mL WFI from vial 1-6.
16. Elution of the product off the SPE using 1.5 mL ethanol from vial 1-7. Collecting the product in vial 1-8, pre-loaded with 16.5 mL sodium ascorbate solution.
17. Using nitrogen pressure, passing the product solution from product vial 1-8 through a 0.22 μm sterile membrane filter into the sterile, filter-vented final product vial.
18. Samples for quality control, sterility testing and reserve sample were withdrawn in a laminar-airflow (LAF) cabinet with grade A environment inside.

### 3.4. Quality Control

Quality control of [^18^F]GP1 is carried out in accordance with common European Pharmacopoeia procedures [25]. Specifications and quality control test methods are listed in Table 1.

Doses are visually examined to be clear and free of particular matter. Radiochemical purity (rcp) is determined by analytical radio HPLC (see Section 3.2). Radiochemical identity is confirmed by co-injection of the GP1 reference compound. Radionuclidic purity is measured with a high purity germanium detector. Radionuclidic identity is measured in an Isomed 2010 dose calibrator. The half-life is calculated from the generated calibration and compared with the known half-life of [^18^F]fluorine (109.8 min). Chemical purity regarding ^19^F-GP1 and unspecified impurities are determined by analytical radio-HPLC (see Section 3.2). Residual solvent analysis is performed by using the Thermo Fisher gas chromatography system Trace 1310, equipped with autoinjector, split/splitless inlet, flame ionization detector (FID) and Permabond OV 1701 column (60 m × 0.313 mm × 1.0 µm). Kryptofix^®^222 levels are analyzed using the established TLC test method [26]. The pH is determined using a calibrated potentiometer. Bacterial endotoxin content is analyzed using the turbidimetric kinetic method. Bioburden, sterility testing and media fill evaluation is performed by GMP-certified laboratory services according to European Pharmacopoeia guidelines [25].

### 3.5. Qualification and Validation

The synthesis module for the synthesis of [^18^F]GP1 was qualified in accordance with EudraLex, Volume 4, GMP guidelines, Annex 15 [27]. [^18^F]GP1 was produced according the GMP guidelines set by European Union (EU GMP Annex 3) [28].

#### 3.5.1. Validation of the Synthesis Procedure

Process validation was performed to confirm that the [^18^F]GP1 production process reliably and reproductively produced a radiopharmaceutical that fulfilled the product specifications until the end of its shelf-life.

For process validation, five consecutive batches of [^18^F]GP1 were produced following the prescribed procedure (see Section 3.3.). The QC tests were performed on the end product at the end of synthesis (EOS), as described in Table 1. Radiochemical purity and pH were also examined at EOS +2 h, +4 h, +6 h, +8 h for the determination of the shelf-life. An additional batch without radioactivity was produced for microbiological verification of bioburden prior to sterile filtration. Validation of the aseptic filling process was performed by media fill trials.

#### 3.5.2. Validation of Analytical Methods

The analytical HPLC method (described in Section 3.2) used for analyzing the radiochemical and chemical purity and for determination of the radiochemical identity of [^18^F]GP1 and the gas chromatography method for analyzing residual solvents were validated following GMP guidelines [27]. The validated parameters for rcp included specificity, limit of detection and quantification, and those for chemical purity were specificity, linearity, repeatability, precision, accuracy, range and detection and quantification limit. The validated parameters for GC were specificity, linearity, repeatability and limit of detection and quantification.

### 3.6. [^18^F]GP1 PET/CT Imaging

PET/CT imaging with the investigational diagnostic radiopharmaceutical [^18^F]GP1 was performed under compassionate use regulations in accordance with the ordinance on Medicinal Products Act (Arzneimittelgesetz—AMG), section 13 (2b), for Compassionate Use of the Federal Institute for Drugs and Medical Devices (BfArM), the European Medicines Agency (EMA)’s Guideline on Compassionate Use of Medicinal Products and under the conditions of the updated Declaration of Helsinki, section 37 (unproven interventions in clinical practice). The compassionate use was approved by the Institutional Review Board (2020/639), and each patient provided written informed consent.

No specific food restrictions were required. [^18^F]GP1 was administered intravenously (<10 nmol, 221–240 MBq). The PET scan acquisition was started 70–90 min after injection of the radiotracer. In both patients, an additional gated acquisition of the heart was performed in list mode. Serial whole-body [^18^F]GP1 PET/CT acquisition covering vertex to thighs was conducted. The PET/CT scans were obtained with a Biograph mCT 128 Flow PET/CT scanner (Siemens Healthcare GmbH, Erlangen, Germany). Unenhanced low-dose CT (120 keV, 35 mAs, CARE Dose, Siemens Healthcare GmbH, Erlangen, Germany) data were reconstructed with a soft-tissue kernel to a slice thickness of 5 mm and 2 mm. PET was acquired in 3-dimensional mode (matrix, 200 × 200) using FlowMotion (Siemens Healthcare GmbH, Erlangen, Germany). The emission data were corrected for randoms, scatter and decay. Reconstruction was performed with ordered-subset expectation maximization using 2 iterations and 21 subsets and Gauss filtration to a transaxial resolution of 5 mm in full width at half maximum. Attenuation correction and anatomical correlation were performed using the unenhanced low-dose CT data.

[^18^F]GP1 PET/CT images were assessed visually and quantitatively by the consensus of 2 experienced physicians who were informed of all standard imaging, clinical and laboratory findings. Lesion detection was done by visual analyses. Lesions with increased uptake in relation to background blood-pool activity measured in the left atrium were regarded as positive thromboembolic foci. These regions of interest were quantitatively assessed by determining the maximum standard uptake value (SUV_max_) and compared with the mean standard uptake value (SUV_mean_) in the left atrium as background. Quantification of clot-to-blood ratio was defined as SUV_max_ of clot uptake / SUV_mean_ of background.

### 3.7. Experimental in Vitro Set Up

Both surgical aortic valves (explanted Edwards Perimount Aortic 2900, size 27mm and an unused Edwards Perimount Aortic 2900, size 23mm) were incubated in [^18^F]GP1/0.9% NaCl solution (20 kBq/mL) for 5 min at room temperature, washed sufficiently with 0.9% NaCl solution and placed in a small plastic box. PET/CT measurement was performed for 900 s with an acquisition speed of 0.5 mm/s.

### 3.8. Western Blot

Material from explanted bioprosthesis was analyzed by western blot using a commercially available antibody (Anti-CD41 antibody (HRP) ab194981 from Abcam, Cambridge, UK) to confirm the presence of the GPIIb/IIIa receptor. Protein preparations were done from apparent thrombi with RIPA-buffer containing protease-inhibitor cocktail (P2714 from Sigma-Aldrich, Taufkirchen, Germany). Protein (30 to 50 µg) were loaded onto a 7.5% SDS-polyacrylamid gel, separated by eletrophoresis and electrotransferred to nitrocellulose sheets. Incubation with the antibody was done overnight at 4 °C using a dilution of 1:1000. For immunodetection, WesternBright™ Quantum (Advansta Inc., CA, USA) was used as HRP-substrate and images were recorded by a CCD-camera system (Alpha Innotech, San Jose, CA, USA).

## 4. Conclusions

Herein, we describe the GMP-compliant synthesis of the novel radiotracer [^18^F]GP1. An improved, robust and reliable fully automated radiosynthesis of [^18^F]GP1 has been developed. The process has been successfully implemented to GMP standard at up to 110 GBq starting activity and has enabled the delivery of [^18^F]GP1 for clinical use. [^18^F]GP1 has been synthesized with decay corrected radiochemical yields of 38 ± 6%, with a radiochemical concentration up to 1900 MBq/mL, molar activities of 952–9428 GBq/µmol and a radiochemical purity >98%. After determination of the optimal reaction conditions, in particular for HPLC separation, adaption of the reaction procedures to clean room conditions, validation of the manufacturing process and the quality control methods, the synthesis of [^18^F]GP1 was successfully implemented to GMP standards and was available for clinical application. Moreover, we provide some proof-of-concept examples for clinical application in the cardiovascular field. [^18^F]GP1 PET/CT may serve as a novel highly sensitive tool in the diagnostic armamentarium of cardiovascular imaging techniques by visualizing active platelet aggregation at the molecular level. [^18^F]GP1 PET/CT may overcome some limitations of current diagnostic approaches. Given its sensitivity and ability to visualize a range of thrombotic lesions, the novel radiotracer [^18^F]GP1 may have broader applications in other cardiovascular diseases associated with platelet aggregation and thrombus formation. As shown in this report, [18]FGP1 PET/CT may potentially prove useful not only for detecting thrombotic complications in patients after bioprosthetic (or mechanical) valve and LVAD implantation but also for monitoring of therapeutic approaches. Further studies will be necessary to evaluate the performance and the incremental diagnostic value of this novel technique when compared to established standard imaging tools.

## Figures and Tables

**Table 1:** Results of the process validation for the clinical preparation of [^18^F]GP1.

Parameter	Acceptance criteria	Method	Batch 1	Batch 2	Batch 3	Batch 4	Batch 5
Appearance	Clear, free of particles	Visual inspection	conforms	conforms	conforms	conforms	conforms
Radiochemical identity	R_t_([^18^F]GP1) = R_t_ (GP1 reference) ± 10%	HPLC/ γ-detector	conforms	conforms	conforms	conforms	conforms
Radiochemical purity	[^18^F]GP1 ≥ 90%	HPLC/ γ-detector	99.6%	99.4%	99.6%	> 99.9%	98.6%
Chemical purity	c_GP1_:([^18^F]GP1 + [^19^F]GP1): ≤ 10.0 µg/dose ^1^	HPLC/UV-detector	0.12 µg/mL	0.34 µg/mL	0.14 µg/mL	0.04 µg/mL	0.46 µg/mL
	Highest unspecific impurities ^2^: ≤1.5 µg/dose ^1^	HPLC/UV-detector	<0.15 µg/mL	<0.15 µg/mL	<0.15 µg/mL	<0.15 µg/mL	<0.15 µg/mL
	K_222_ ≤ 50 µg/mL	TLC	<50 µg/mL	<50 µg/mL	<50 µg/mL	<50 µg/mL	<50 µg/mL
	Acetonitrile: ≤410 µg/mL	GC	<410 µg/mL	<410 µg/mL	<410 µg/mL	<410 µg/mL	<410 µg/mL
	Ethanol: ≤79,000 µg/mL	GC	<79,000 µg/mL	<79,000 µg/mL	<79,000 µg/mL	<79,000 µg/mL	<79,000 µg/mL
Radionuclidic purity	≥99.9%	HPGe detector	>99.9%	>99.9%	>99.9%	>99.9%	>99.9%
Radionuclidic identity	Deviation: <±4.5%	Dose calibrator	−0.49%	−2.56%	−0.27%	2.46%	0.01%
pH	4.0 ≤ pH ≤ 8.5	Potentiometry	7.7	7.5	7.4	7.7	7.2
Bacterial endotoxins	≤17.5 EU/mL	Ph. Eur.	<0.25 EU/mL	<0.25 EU/mL	<0.25 EU/mL	<0.25 EU/mL	<0.25 EU/mL
Sterile filter integrity	≥3500 mbar	Bubble point test	conforms	conforms	conforms	conforms	conforms
Sterility ^3^	sterile	Ph. Eur.	sterile	sterile	sterile	sterile	sterile
Strength at EOS	c_EOS_ ≥ 50 MBq/mL	Dose calibrator	612 MBq/mL	676 MBq/mL	721 MBq/mL	788 MBq/mL	1330 MBq/mL
Specific molar activity		$\mathrm{specific}\;\mathrm{molar}\;\mathrm{activity}=\frac{c_{\mathrm{EOS}}\times M_{GP1}}{c_{\mathrm{GP}1}\times 1000}$;	2440 GBq/µmol	952 GBq/µmol	2465 GBq/µmol	9428 GBq/µmol	1388 GBq/µmol
Radiochemical yield (d.c.) ^4^			34.0%	32.0%	37.2%	43.6%	35.3%
Synthesis time			69 min	72 min	76 min	68 min	76 min

^1^ Single dose volume ≤ 10 mL.^2^ Assuming an identical UV absorption as GP1.^3^ Test conducted post release. ^4^ Starting activity: 50–110 GBq.

**Table 2:** Preparation steps and solutions for the synthesis of [^18^F]GP1.

Vial No	Preparation Steps
1-1	1.5 mL of K_222_/K_2_CO_2_ solution (1.0 mg K_2_CO_3_, 5.0 mg K_222_, 0.25 mL WFI, 1.25 mL acetonitrile)
1-3	1 mL of anhydrous acetonitrile
1-2	7 mg GP1 precursor dissolved in 0.5 mL anhydrous acetonitrile
1-5	3 mL WFI + 0.25 mL 3 N NaOH
1-4	1.0 mL 1N HCl
1-6	5 mL WFI
1-7	1.5 mL ethanol
1-8	16.5 mL sodium ascorbic acid solution (27.3 mg/mL in WFI) (Product)
2-1	70 mL WFI (Collect)

## Data Availability

The data presented in this study are available on request from the corresponding author. The data are not publicly available due to privacy.

## Supplementary Materials

The following are available online at https://www.mdpi.com/article/10.3390/ph14080739/s1. Figure S1: Western blot of endocarditic material. Video S1: Rotating, 3-dimesional maximum intensity projection (MIP) PET image reconstruction of patient # 1. Video S2: Rotating, 3-dimesional maximum intensity projection (MIP) PET image reconstruction of patient # 2. Video S3: Rotating, 3-dimesional maximum intensity projection (MIP) PET image reconstruction of endocarditic bioprosthesis. Video S4: Rotating, 3-dimesional maximum intensity projection (MIP) PET image reconstruction of unused bioprosthesis.
