# Supplementary material for: GMP-Compliant Radiosynthesis of [18F]GP1, a Novel PET Tracer for the Detection of Thrombi
Source: Pharmaceuticals (Basel). 2021 Jul 28;14(8):739. doi: 10.3390/ph14080739 (PMC8399972; doi:10.3390/ph14080739)
Supplement: Supplementary file 1 [file pharmaceuticals-14-00739-s001.zip › Supporting Information.pdf]

Supporting Information:

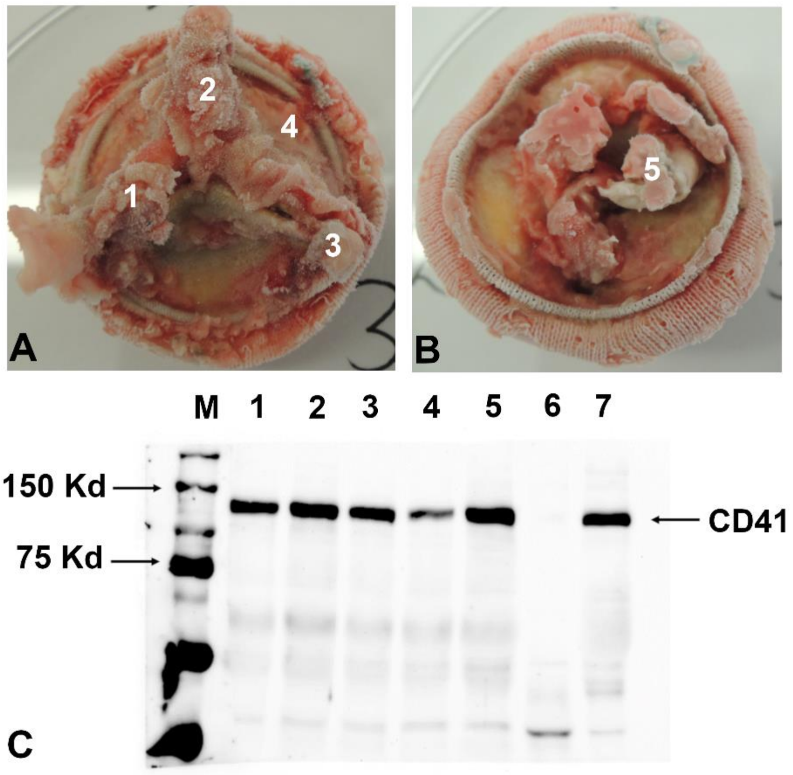

Figure S1. Western blot of endocarditic material on explanted bioprothesis: (**a**) Bottom view of frozen explanted bioprosthesis, numbers 1-3 indicating the positions of material taken for western blot; (**b**) Top view of frozen explanted bioprosthesis, numbers 4-5 indicating the positions of material taken for western blot; (**c**) Western blot of endocarditic material (numbers 1-5) confirmed the presence of GPIIb/IIIa receptor, line 6: negative control, line 7: positive control with GPIIb/IIIa positive material.

Video S1: Rotating, 3-dimesional maximum intensity projection (MIP) PET image reconstruction of patient # 1.

Video S2: Rotating, 3-dimesional maximum intensity projection (MIP) PET image reconstruction of patient # 2.

Video S3: Rotating, 3-dimesional maximum intensity projection (MIP) PET image reconstruction of endocarditic bioprosthesis.

Video S4: Rotating, 3-dimesional maximum intensity projection (MIP) PET image reconstruction of unused bioprosthesis.
