# Supplementary figures and images for: GMP-Compliant Radiosynthesis of [18F]GP1, a Novel PET Tracer for the Detection of Thrombi
Source: Pharmaceuticals (Basel). 2021 Jul 28;14(8):739. doi: 10.3390/ph14080739 (PMC8399972; doi:10.3390/ph14080739)

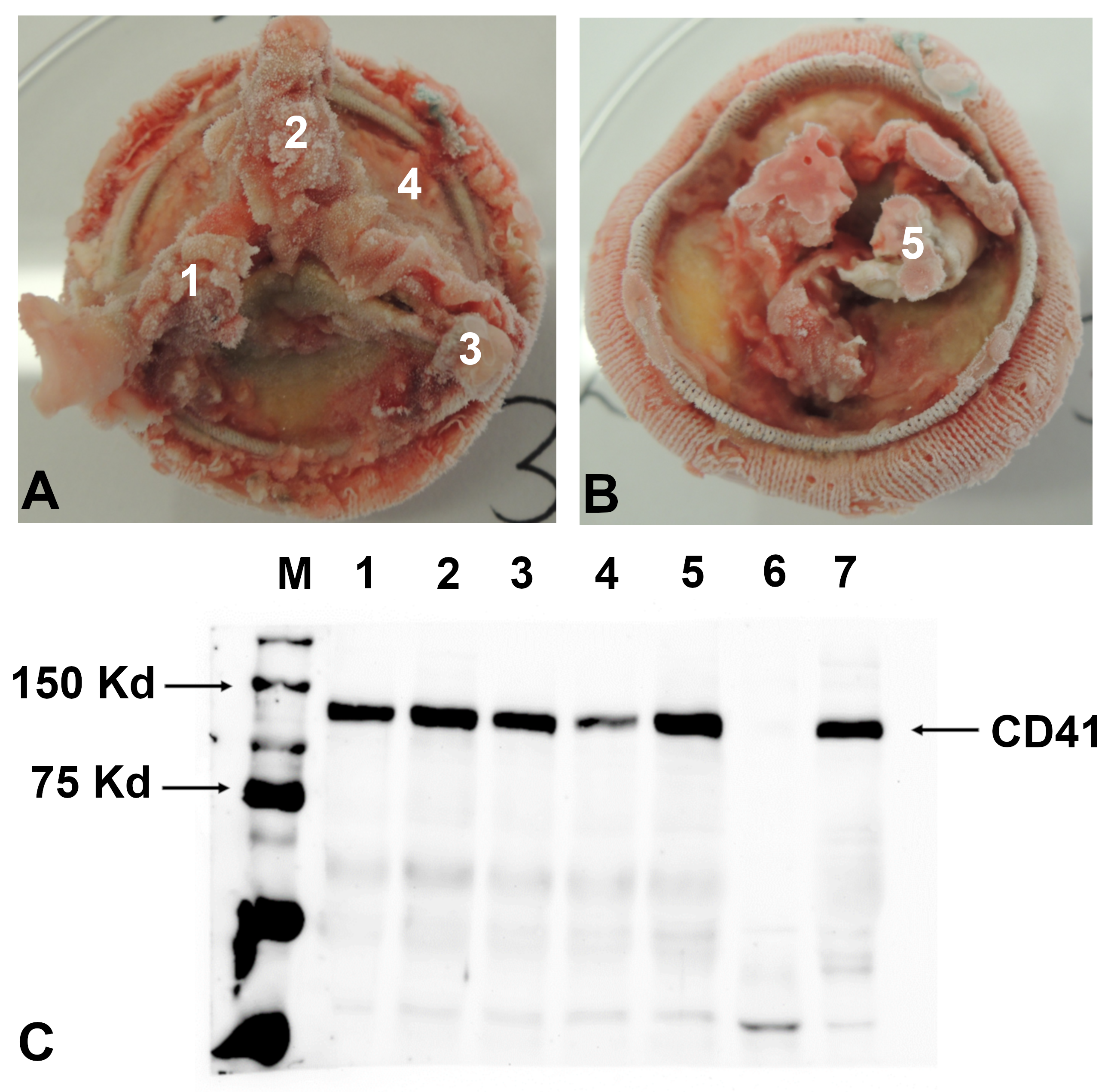

Supplement: Supplementary file 1 [file pharmaceuticals-14-00739-s001.zip › Figure S1_Western blot.tif]
